# The Effects of Tributyrin on Immune Function, Antioxidant Capacity, and Metabolomics in Young Pigeons

**DOI:** 10.3390/ani16101547

**Published:** 2026-05-18

**Authors:** Run Wu, Lihuan Deng, Haiying Li, Yingying Yao, Yingping Wu, Qingqing Lu, Gaoyun You, Tinghao Jiang

**Affiliations:** College of Animal Science, Xinjiang Agricultural University, Urumqi 830052, China; 18555384822@163.com (R.W.); dlh2498217132@163.com (L.D.); yyy1234560317@163.com (Y.Y.); wyp_941208@163.com (Y.W.); 17590813280@163.com (Q.L.); 17628603149@163.com (G.Y.); jth-007@163.com (T.J.)

**Keywords:** tributyrin, young pigeon, immune function, antioxidant capacity, metabolomics

## Abstract

Tributyrin, a short-chain fatty acid derivative, has been shown to hold potential in improving intestinal health in livestock and poultry. However, its multidimensional effects on the health of meat pigeons, particularly during the young pigeon stage, remain unclear. This study aimed to investigate the comprehensive effects of dietary tributyrin supplementation on the growth, health status, intestinal function, and metabolic profile of young pigeons. A total of 100 healthy 29-day-old White King pigeons, with half male and half female, were randomly divided into a control group (fed a basal diet) and a treatment group (fed a basal diet supplemented with 1500 mg/kg tributyrin) for a 35-day trial. The results showed that compared with the control group, young pigeons in the treatment group had significantly reduced serum triglyceride levels, alanine aminotransferase activity, and concentrations of pro-inflammatory cytokines (TNF-α, IL-6), along with significantly increased levels of high-density lipoprotein, immunoglobulin G, total antioxidant capacity, and glutathione peroxidase activity. Concurrently, the villus height-to-crypt depth ratio in the jejunum and ileum was significantly elevated, indicating improved intestinal morphological structure. Untargeted metabolomics analysis further revealed significant changes in the relative abundances of 13 key differential metabolites (e.g., L-carnitine, pyridoxamine, indoleacetic acid) in the small intestinal contents of the treatment group. These metabolites were mainly enriched in metabolic pathways such as 2-oxoCarboxylic acid metabolism, tryptophan metabolism, and vitamin B6 metabolism. In conclusion, dietary supplementation with 1500 mg/kg tributyrin can exert multifaceted beneficial effects on young pigeon health by improving lipid metabolism, enhancing immune and antioxidant functions, optimizing intestinal structure, and regulating the local metabolic network. This study provides a theoretical basis for the application of tributyrin as a functional additive in the green and healthy production of meat pigeons.

## Simple Summary

Tributyrin, a novel feed additive, has been shown to hold great potential in enhancing gut health and immune function in animals such as pigs and chickens. However, its effects on meat pigeons, especially during the young pigeon stage, remain unclear. This study systematically evaluated the multidimensional impacts of dietary supplementation with 1500 mg/kg tributyrin on young pigeons. The results indicated that this supplementation significantly increased serum immunoglobulin G levels and total antioxidant capacity, optimized the morphological structure of the jejunum and ileum, and regulated several key metabolic pathways in the small intestinal contents, which are associated with lipid, amino acid, and vitamin metabolism. These findings clarify the beneficial effects of tributyrin in promoting the overall health and nutrient utilization of young pigeons, thereby providing a new scientific basis for the development of green feeding strategies in pigeon production.

## 1. Introduction

With the continuous optimization of livestock and poultry breeding structure in China, meat pigeons have become the fourth largest poultry industry due to their advantages of high protein, low fat, and delicious meat [1]. In recent years, the intensive farming level of meat pigeons has been continuously improved, but there are still two key problems restricting the healthy development of the industry: first, the nutritional standard system of meat pigeons is not perfect, and the research and application of green feed additives are insufficient [2]; second, the frequent occurrence of intestinal diseases and immune stress under high-density feeding leads to slow growth and poor health of young pigeons [3]. Therefore, it is of great practical significance to develop safe, efficient, and residue-free green feed additives to improve the immune function, antioxidant capacity, and intestinal health of young pigeons.

As a new type of green feed additive, tributyrin can be slowly decomposed into butyric acid in the animal intestinal tract, which provides energy for intestinal epithelial cells, repairs intestinal mucosal barrier, regulates intestinal flora balance, enhances body immunity and antioxidant capacity, and has no residue and no drug resistance risk [4,5]. At present, tributyrin has been widely studied in pigs, broilers, sheep, and other livestock and poultry. Studies have shown that dietary supplementation of tributyrin can significantly improve serum antioxidant indexes, immunoglobulin content, and intestinal morphology of broilers and Hu sheep [6,7]. However, the research on tributyrin in meat pigeons is still blank, especially the systematic evaluation of its effects on immune function, antioxidant capacity, intestinal morphology, and intestinal metabolomics of young pigeons has not been reported.

In view of this, this study took 29-day-old White King young pigeons as the research objects and added 1500 mg/kg tributyrin to the diet to explore its effects on serum biochemical indexes, immune function, antioxidant capacity, intestinal tissue morphology, and intestinal metabolomics of young pigeons. The purpose of this study is to clarify the mechanism of tributyrin in improving the health of young pigeons and to provide a theoretical basis and technical support for the application of tributyrin as a green feed additive in meat pigeon breeding.

## 2. Materials and Methods

### 2.1. Ethical Statement

All the experimental protocols were approved by the Animal Ethics Committee of Xinjiang Agricultural University (Approval Number: 2023008).

All procedures were performed in accordance with animal welfare guidelines to minimize suffering.

### 2.2. Experimental Design

Tributyrin (TB) was provided by Guangdong Yidali Biotechnology Co., Ltd. (Zhuhai, China). A total of 100 healthy 29-day-old White King pigeons with uniform body weight and a balanced sex ratio (half male and half female) were used in this experiment. All birds were reared at the experimental farm of Luohai Pigeon Industry Development Co., Ltd., located in Moyu County, Hotan Prefecture, Xinjiang, China. The pigeons were randomly allocated into two groups as follows: a control group (CK), which were fed a basal diet, and a tributyrin treatment group (B), which were fed the basal diet supplemented with 1500 mg/kg tributyrin. The dose of 1500 mg/kg tributyrin was selected according to our preliminary trial data and previously published literature on poultry [8].

Each group contained 5 replicates with 10 pigeons per replicate. The total experimental period lasted 35 days, including a 7-day adaptation phase and a 28-day formal experimental phase. During the entire formal trial, the CK group was fed the basal diet, while group B was fed the same diet supplemented with 1500 mg/kg tributyrin. The basal diet was formulated to meet the nutritional requirements of growing White King pigeons, following the farm’s feeding specifications and widely accepted research standards. The detailed ingredients and calculated nutrient levels of the basal diet are shown in Table 1.

### 2.3. Feeding Management

During the entire experimental period, the test pigeons were housed in two-tier cages, with each cage measuring 40 cm × 50 cm × 35 cm (length × width × height) with 3 pigeons per cage. Feed was provided ad libitum using manual feeders, and drinking water was supplied artificially at fixed times every day. Health sand was available at all times. The ambient temperature in the pigeon house was maintained at 20–25 °C, and the relative humidity was controlled at 55–65%. Natural lighting was provided through transparent windows and ventilation openings in the house, following the natural photoperiod. The pigeon house was well-ventilated and manually cleaned three times a day. Throughout the trial, the spirit, appetite, activity, feather condition, and fecal status of the pigeons were observed and recorded daily. All other feeding and management measures were carried out in accordance with conventional procedures.

### 2.4. Sample Collection

On the 28th day of the trial’s formal period, 16 meat pigeons were randomly selected from each group. A total of 5 mL of blood was collected from the subcutaneous vein and then centrifuged at 2500 r/min for 10 min. The serum was aliquoted into 1.5 mL centrifuge tubes and stored at −20 °C for the detection of serum parameters. Subsequently, they were euthanized and samples were collected. All pigeons were humanely euthanized by cervical dislocation following standard animal welfare guidelines. About 2 cm of tissue was intercepted from the middle segment of the duodenum, jejunum, and ileum respectively. The duodenal, jejunal, and ileal tissue samples were rinsed with phosphate-buffered saline (PBS) buffer solution and immediately fixed in 4% paraformaldehyde solution for the observation of small intestinal tissue morphology; the contents of the small intestine were collected and stored at −80 °C for the analysis of intestinal metabolites and metabolic pathways.

### 2.5. Determination of Serum Parameters

We determined the serum biochemical indicators: total protein (TP), albumin (ALB), total cholesterol (TC), triglycerides (TG), alanine aminotransferase (ALT), aspartate aminotransferase (AST), high-density lipoprotein (HDL), and low-density lipoprotein (LDL) content; we determined the serum immune indicators: immunoglobulin G (IgG), IgA, IgM content, interleukin-2 (IL2), IL-6, IL-12, and TNF-α content; and we determined the serum oxidative and antioxidant indicators: total antioxidant capacity (T-AOC), superoxide dismutase (SOD), glutathione peroxidase (GSH-Px) activity, and malondialdehyde (MDA) content. All above serum parameters were detected using commercial assay kits provided by Sichuan Weil Testing Technology Co., Ltd., Chengdu, China and the operation was strictly performed according to the manufacturer’s instructions.

### 2.6. Observation of Intestinal Morphology

The duodenal, jejunal, and ileal tissues were taken out from paraformaldehyde fixative, followed by tissue blocking, dehydration, and paraffin embedding. Serial sections were prepared at a thickness of 5 μm using a microtome (Leica, Wetzlar, Germany). The sections were stained with hematoxylin–eosin (HE) and observed under a light microscope (Olympus, Tokyo, Japan). The histological images were collected and initially processed with Motic Images Advanced 3.2 software (Motic China Group Co., Ltd., Xiamen, China). Villus height (VH), crypt depth (CD), and VH/CD ratio were further measured and calculated using ImageJ (version 1.53) software.

### 2.7. Metabolomic Analysis of Intestinal Contents

#### 2.7.1. Sample Preprocessing for Liquid Chromatography–Mass Spectrometry (LC-MS)

① Take 100 mg of liquid nitrogen-frozen and ground tissue samples and place them in an EP tube. Add 500 μL of 80% methanol–water solution. ② Vortex and shake, and then incubate on ice for 5 min. Centrifuge at 15,000× *g* for 20 min at 4°C. ③ Take a certain amount of the supernatant and dilute it with mass spectrometry-grade water to a methanol content of 53%. ④ Centrifuge at 15,000× *g* for 20 min at 4°C, collect the supernatant, and inject it into LC-MS for analysis. The LC-MS/MS analysis was provided by Nuoheshizhong Technology Co., Ltd. (Beijing, China).

#### 2.7.2. Chromatographic Conditions

Column: HypesilGoldcolumn (C18) (particle size 1.9 μm, specification 100 mm × 2.1 mm); sample injection volume: 3 μL; column temperature: 40°C. In positive ion mode, the mobile phase A is 0.1% formic acid, and B is methanol; in negative ion mode, the mobile phase A is 5 mM ammonium acetate (pH 9.0), and B is methanol. The gradient elution program is shown in Table 2.

#### 2.7.3. Mass Spectrometry Conditions

For each sample, positive and negative ion mode detection was performed using electrospray ionization (ESI, Thermo Fisher Scientific, Waltham, MA, USA). The scan range was set at m/z: 100–1500; ESI source conditions: spray voltage: 3.5 kV; sheath gas flow rate: 35 psi; auxiliary gas flow rate: 10 L/min; ion transmission tube temperature: 320 °C; ion introduction radio frequency level: 60; auxiliary gas heater temperature: 350 °C; polarity: positive (positive), negative (negative); the MS/MS secondary scan was a data-dependent scan.

### 2.8. Data Processing and Statistics

The independent sample *t*-test was conducted on the experimental data using SPSS 20.0 software. Before the analysis, data normality was tested. Normally distributed data were analyzed through independent sample *t*-test, while non-normally distributed data were analyzed by the non-parametric Mann–Whitney U test. The results were expressed as “mean ± standard deviation”. A *p*-value below 0.05 indicated a significant difference, a *p*-value below 0.01 indicated an highly significant difference, and a *p*-value greater than 0.05 indicated no significant difference. In the metabolomics data analysis, Compound Discoverer 3.1 software was used for peak extraction and area quantification, and metaX software (version 1.0) was used for principal component analysis (PCA) and partial least squares–discriminant analysis (PLS-DA). The criteria for screening differential metabolites were that the variable projection importance (VIP) was greater than 1, the *p*-value was lower than 0.05, and the fold change (FC) was greater than 1.2 or lower than 0.833. The volcano plot and bubble plot were generated using R software (Version 4.2.1, R Core Team, Vienna, Austria). Pearson correlation analysis was performed to evaluate the correlations among the measured physiological and biochemical indices.

## 3. Results

### 3.1. The Influence of Tributyrin on the Biochemical Indicators of Pigeon Serum

As shown in Table 3, compared with the CK group, the B group showed highly significantly lower ALT activity (35.04%, *p* < 0.01), significantly lower TG content (18.52%, *p* < 0.05), and significantly higher HDL concentration (20.13%, *p* < 0.05). No significant differences were observed in other serum biochemical indices (*p* > 0.05).

### 3.2. The Influence of Tributyrin on the Immune Indicators of Pigeon Serum in Young Pigeons

As presented in Table 4, dietary tributyrin significantly increased serum IgG content by 35.04% (*p* < 0.01). Meanwhile, TNF-α and IL-6 contents were significantly decreased by 17.33% and 21.06%, respectively (*p* < 0.05). Other immune indices showed no significant differences between groups (*p* > 0.05).

### 3.3. The Effect of Tributyrin on the Antioxidant Indices of Young Pigeon Serum

As shown in Table 5, compared with the CK group, the T-AOC and GSH-Px activities in the B group were highly increased by 88.04% and 22.08% respectively (*p* < 0.01), while there were no significant differences in the other indicators among the groups (*p* > 0.05).

### 3.4. The Effect of Tributyrin on the Intestinal Morphology of Young Pigeons

**Figure 1:**
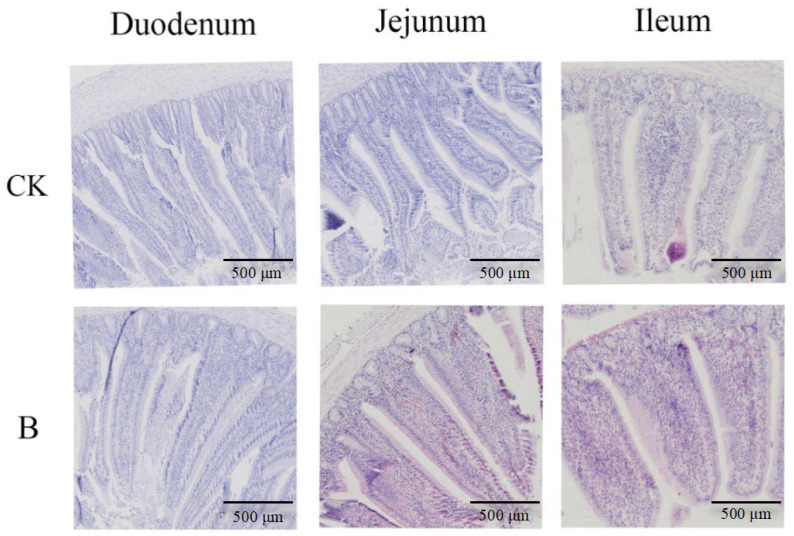
Tissue sections of the duodenum, jejunum, and ileum of young White King pigeons (HE staining, ×40 magnification).

As shown in Table 6, compared with the CK group, the VH/CD ratio of the jejunum and ileum in group B was significantly increased by 37.00% and 34.97% respectively (*p* < 0.01), the CD in the jejunum was highly significantly reduced by 20.43% (*p* < 0.01), the VH in the jejunum was significantly increased by 9.20% (*p* < 0.05), the CD in the ileum was significantly reduced by 21.13% (*p* < 0.05), and there were no significant differences in other indicators among the groups (*p* > 0.05). In the CK group, the villi arrangement of the duodenum, jejunum, and ileum was relatively loose, and in some areas, the villi were slightly broken or detached, and the crypt structure was slightly irregular. In group B, the villi arrangement of the small intestine was dense and regular, the morphology was complete, there was no obvious breakage or detachment, the crypt structure was regular, no obvious tissue damage or inflammatory infiltration was observed, and the intestinal mucosal barrier structure was more complete (Figure 1).

### 3.5. The Effect of Tributyrin on the Metabolic Profile of Intestinal Contents in Young Pigeons

#### 3.5.1. Sample Quality Control (QC) Analysis

**Figure 2:**
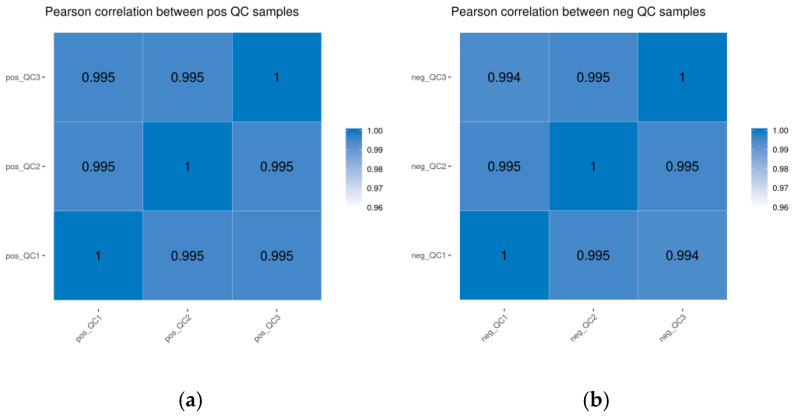
Overlay plots of quality control (QC) samples and their pearson correlation heatmaps ((**a**): positive ion mode; (**b**): negative ion mode).

The Pearson correlation coefficient matrices of the QC samples under both positive and negative ion detection modes were analyzed. The correlation coefficients of the QC samples were all higher than 0.994, indicating that the stability of the entire detection process was good and the data quality was high (Figure 2).

#### 3.5.2. Principal Component Analysis of All Samples

**Figure 3:**
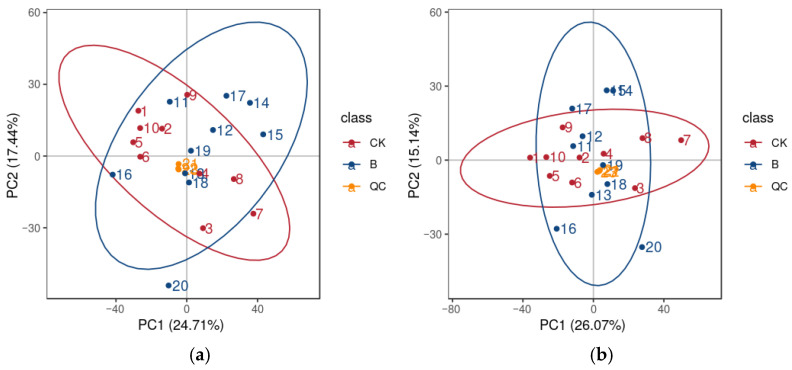
Principal component analysis (PCA) score plots of all samples ((**a**): positive ion mode; (**b**): negative ion mode).

The peaks obtained from all the test samples and QC samples were subjected to PCA. The distribution of the QC samples clustered together, further demonstrating excellent stability of the entire method and high quality of the metabolite identification data (Figure 3).

#### 3.5.3. Least Squares Discriminant Analysis of the Contents of the Small Intestine of Chicks

**Figure 4:**
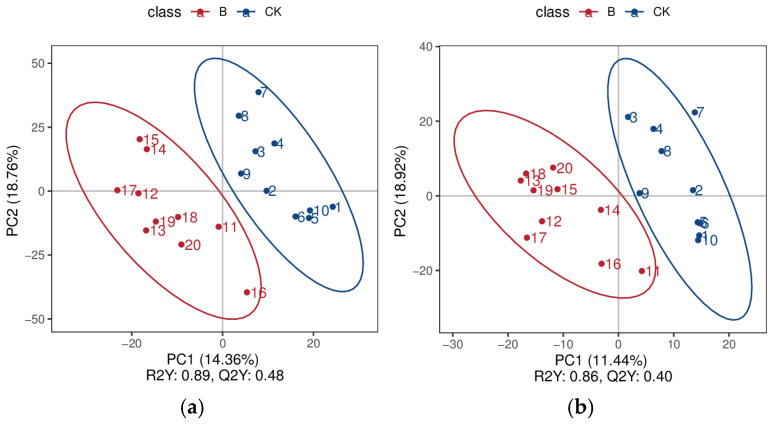
Partial least squares–discriminant analysis (PLS-DA) score plots of small intestinal contents samples from the CK and B groups ((**a**): positive ion mode; (**b**): negative ion mode).

As shown in Figure 4, the R^2^Y value of the PLS-DA positive ion model is 0.89, and Q^2^ is 0.48; the R^2^Y value of the PLS-DA negative ion model is 0.86, and Q^2^Y is 0.40. Both R^2^Y and Q^2^Y are greater than 0.4, indicating that this model has high explanatory power and predictive ability, and the model is stable and reliable. The separation of the two groups of data under the positive and negative ion modes is good, and it can effectively distinguish the samples of the CK group and the B group.

**Figure 5:**
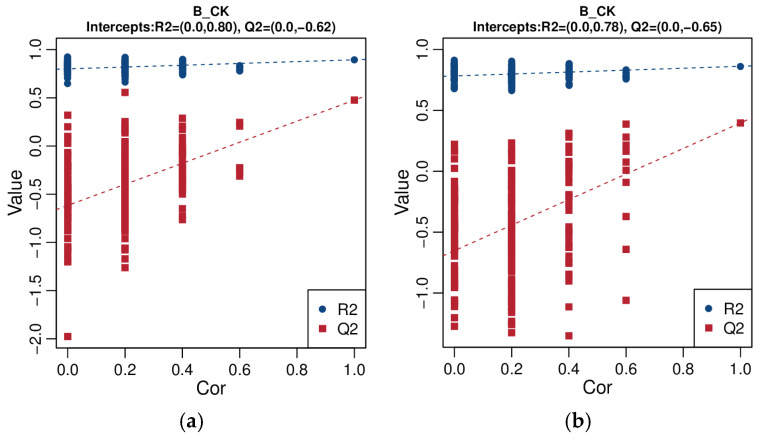
Permutation test plots of the PLS-DA analysis models ((**a**): positive ion mode; (**b**): negative ion mode).

To further verify whether the PLS-DA model is overfitting, a random permutation test was conducted. As shown in Figure 5, the Q^2^ values for the positive ion model and the negative ion model were −0.62 and −0.65 respectively, and the Q^2^ intercepts were both less than 0. This indicates that there is no “overfitting” phenomenon in the model, suggesting that this model can well describe the samples and can be used for the subsequent analysis of differential metabolites screening.

#### 3.5.4. Analysis of Differentially Abundant Metabolites in the Small Intestine of Young Pigeons

**Figure 6:**
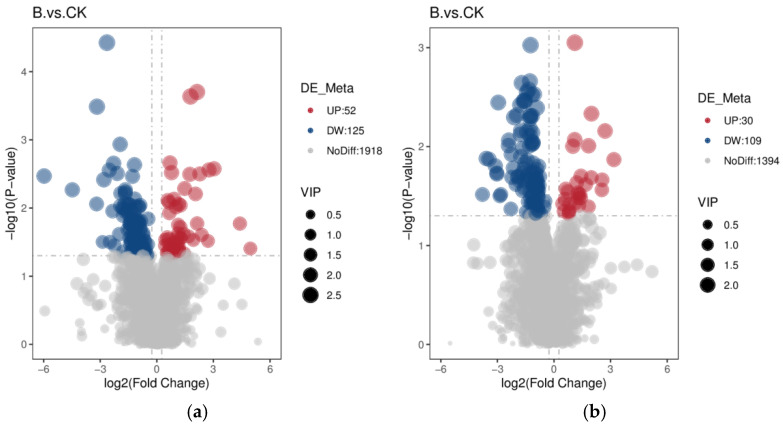
Volcano plots of metabolites in small intestinal contents from the CK and B groups ((**a**) positive ion mode; (**b**) negative ion mode).

Non-targeted metabolite (LC-MS/MS) analysis of the intestinal contents of young pigeons was conducted. In the positive ion mode (Figure 6a), a total of 2095 metabolites were identified, among which 177 were significantly different. There were 52 upregulated metabolites and 125 downregulated metabolites. In the negative ion mode (Figure 6b), a total of 1533 metabolites were identified, and 139 were significantly different. There were 30 upregulated metabolites and 109 downregulated metabolites.

From Table 7 and Table 8, it can be seen that in both positive and negative ion modes, a total of 13 key differential metabolites were identified. Specifically, in the positive ion mode, the relative contents of thiobenzamide S, (7E,13Z,18R,20Z)-felixinin, allyl methyl sulfone, N-methylated cantharidin, tryptophan-threonine-histidine, and pyridoxamine metabolites were significantly increased (FC > 1.2); the relative contents of protoporphyrin IX, L-carnitine, myriocin, and 7,8-dihydrobiopterin were significantly decreased (FC < 0.833). In the negative ion mode, the relative content of indoleacetic acid metabolites was significantly increased (FC > 1.2); the relative contents of N-alpha-acetyl-L-ornithine and phytanic acid were significantly decreased (FC < 0.833).

#### 3.5.5. Metabolic Pathway Enrichment Analysis of Differential Metabolites in the Intestinal Contents of Young Pigeons

**Figure 7:**
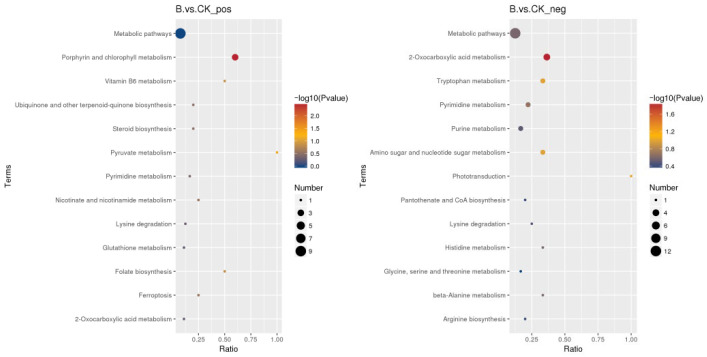
Pathway enrichment bubble plots of differential metabolites in small intestinal contents.

As shown in Figure 7, the differential metabolites were significantly enriched in multiple metabolic pathways. Among them, they were highly significantly enriched in the 2-keto acid metabolism pathway and the porphyrin and chlorophyll metabolism pathways (*p* < 0.01) and significantly enriched in the vitamin B6 metabolism, folic acid biosynthesis pathway, tryptophan metabolism, and amino sugar and nucleotide sugar metabolism synthesis pathways (*p* < 0.05). Among these pathways, 2-oxocarboxylic acid (2-keto acid) metabolism is closely related to energy and nutrient utilization in young pigeons, supporting efficient metabolic balance. Tryptophan metabolism participates in regulating anti-inflammatory and immune responses, which is consistent with the decreased serum TNF-α and IL-6 levels in this study. Vitamin B6 metabolism provides key cofactors for antioxidant and immune functions, directly explaining the increased T-AOC, GSH-Px, and IgG observed in this study. These pathways are highly physiologically relevant and clearly reveal the mechanism by which tributyrin improves metabolism, immunity, antioxidant capacity, and intestinal health in young pigeons.

## 4. Discussion

### 4.1. The Effects of Tributyrin on the Biochemical Indicators of Young Pigeon Serum

Serum biochemical indicators serve as important references for evaluating the metabolic status and health condition of livestock and poultry [9]. An increase in total protein and albumin contents indicates improved absorption and utilization of feed nutrients, which is conducive to enhancing immune function and promoting tissue protein deposition [10]. Total cholesterol content reflects the process of fat metabolism and transformation, while triglycerides, as the main form of energy storage in the body, are closely associated with energy metabolism [11]. High-density lipoprotein acts as a “cholesterol scavenger” by transporting peripheral cholesterol to the liver for metabolism [12], whereas low-density lipoprotein is related to cholesterol deposition in the body; together, these two lipoproteins constitute a comprehensive evaluation system for lipid metabolism [13]. Alanine aminotransferase (ALT) and aspartate aminotransferase (AST) are mainly distributed in hepatocytes; when the liver is damaged or its metabolism is abnormal, the serum activities of these two enzymes will increase significantly [14].

In the present study, dietary supplementation with tributyrin significantly reduced serum triglyceride levels and ALT activity, while increasing high-density lipoprotein (HDL) levels in young pigeons. Jin et al. [15] reported that adding 1000 mg/kg tributyrin and oxymatrine to the diet could significantly increase serum HDL content in 21-day-old broilers. Lu et al. [16] found that supplementing the basal diet of 49-day-old broilers with 1000–2000 mg/kg tributyrin significantly increased serum HDL content and decreased serum triglyceride content. Yang et al. [17] also demonstrated that adding 0.08% and 0.15% tributyrin to the diet of weaned piglets could significantly reduce serum ALT activity.

Integrating our results with previous findings, we confirm that tributyrin exerts consistent beneficial effects on lipid metabolism and liver health, and this study provides new evidence for these effects in young pigeons. Collectively, these studies have verified that tributyrin exerts positive effects on lipid metabolism and liver protection in livestock and poultry. In the current experiment, supplementing the diet with 1500 mg/kg tributyrin significantly reduced serum triglyceride content and ALT activity, while significantly increasing serum HDL levels in young pigeons. This is consistent with the results of previous studies, indicating that tributyrin can effectively improve the lipid metabolism capacity of young pigeons and reduce the metabolic burden on the liver.

In conclusion, the regulatory effect of dietary tributyrin on serum biochemical indicators of young pigeons further confirms its potential as a green and efficient feed additive. By improving lipid metabolism and protecting liver function, tributyrin can help maintain the normal physiological state of young pigeons, laying a solid foundation for their healthy growth. This finding not only enriches the theoretical basis for the application of tributyrin in meat pigeon production but also provides practical guidance for the development of green and antibiotic-free feeding strategies in the meat pigeon industry.

### 4.2. The Effects of Tributyrin on Immune Indicators in Young Pigeon Serum

Serum immune indicators are important parameters for evaluating the immune function of the body. Immunoglobulins (IgG, IgM, and IgA) and cytokines (e.g., IL-10, TNF-α, IL-1β, and IL-6) jointly maintain the body’s immune balance through antigen recognition and inflammatory regulation [18]. IgG, as the main immunoglobulin in the serum, plays crucial roles in anti-infection and virus neutralization [19]. IgM possesses strong antigen-binding capacity and is involved in the early immune response to antigen exposure [20]. IgA can clear antigens and prevent pathogen invasion [21]. Pro-inflammatory factors such as TNF-α, IL-1β, and IL-6 can activate the immune system to eliminate pathogens, while anti-inflammatory factors such as IL-10 can mitigate inflammation and restore the body’s normal physiological state [22]. In general, higher serum levels of immunoglobulins and anti-inflammatory factors, along with lower levels of pro-inflammatory factors, indicate a stronger immune function of the body [23].

Our results showed that dietary tributyrin significantly increased serum IgG levels and decreased serum TNF-α and IL-6 levels in young pigeons. Liu et al. [24] reported that supplementing the basal diet of broilers with 500 mg/kg coated sodium butyrate could significantly reduce serum IL-6 content, suggesting that short-chain fatty acids can improve broiler immune function and alleviate inflammatory responses. Miao et al. [25] demonstrated that after 4 weeks of supplementing *Escherichia coli*-infected mice with a combination of plant essential oil and tributyrin, serum TNF-α content significantly decreased while serum IgG content significantly increased. Liang et al. [5] also found that dietary tributyrin supplementation could regulate the immune function of animals. The results of this experiment showed that adding tributyrin to the diet significantly increased serum IgG content and reduced TNF-α and IL-6 levels in young pigeons, which is consistent with the aforementioned research findings.

Combined with these reports, our findings further demonstrate that tributyrin enhances humoral immunity and suppresses inflammatory responses in a species-specific manner in young pigeons. This beneficial effect might be associated with the ability of tributyrin to promote humoral immune function and inhibit excessive inflammatory responses, thereby improving the overall immune status of young pigeons.

### 4.3. The Effects of Tributyrin on Antioxidant Indexes in Young Pigeon Serum

During normal life activities, animals continuously generate free radicals, which require a complete antioxidant system to protect tissues, organs, and cells from oxidative damage [26]. Total antioxidant capacity (T-AOC) reflects the overall antioxidant level of the body [27]. Superoxide dismutase (SOD) can catalyze the conversion of superoxide anions into hydrogen peroxide (H_2_O_2_) and oxygen (O_2_), thereby reducing cellular oxidative damage [28]. Glutathione peroxidase (GSH-Px) can decompose hydrogen peroxide through the redox reaction of glutathione, converting it into harmless substances. It works synergistically with SOD to maintain cellular redox balance and reduce the risk of oxidative damage [29]. Malondialdehyde (MDA) is the main product of lipid peroxidation and exhibits cytotoxicity; its content can directly reflect the degree of lipid peroxidation and cellular damage in the body, and its level increases significantly under oxidative stress conditions [30].

In this experiment, dietary supplementation with tributyrin significantly increased the serum T-AOC level and GSH-Px activity in young pigeons, which is consistent with the research findings of Wang et al. and Wang et al. [31,32]. This indicates that tributyrin can enhance the body’s antioxidant function, scavenge free radicals, and alleviate oxidative damage, thereby improving the overall antioxidant capacity of young pigeons.

### 4.4. The Effect of Tributyrin on the Intestinal Morphology of Young Pigeons

Villus height (VH), crypt depth (CD), and the villus–crypt ratio (VH/CD) are important indicators for assessing intestinal health and core parameters for evaluating an animal’s nutrient absorption capacity [33]. A higher villus height can expand the contact area between the intestinal mucosa and chyme, thereby enhancing digestive and nutrient absorption capabilities [34]. Meanwhile, it increases the number of single-layer columnar cells in the intestinal wall, which facilitates nutrient absorption and transport and stimulates the secretion of intestinal enzymes to further improve nutrient absorption function. A shallower crypt depth indicates higher maturity of intestinal epithelial cells and stronger ability to secrete digestive juices [35]. Therefore, an increased villus–crypt ratio is generally considered beneficial for nutrient absorption in animals.

Ismael et al. [36] reported that supplementing the diet with a complex of 500 g/t tributyrin (40%) + copper + essential oil could significantly increase the jejunal and ileal VH of 35-day-old broilers. Shang et al. [37] found that adding 1000 g/t of a mixture of tributyrin and lauric acid monoglyceride to the diet significantly reduced the jejunal CD of 21-day-old and 42-day-old broilers and significantly increased the jejunal and ileal VH/CD of 42-day-old broilers. Cui et al. [38] demonstrated that supplementing 750 mg/kg and 1000 mg/kg sodium butyrate could significantly increase the jejunal VH of 21-day-old and 42-day-old broilers, while 1000 mg/kg sodium butyrate could significantly reduce the jejunal CD of 42-day-old broilers.

These consistent effects across different animal species suggest that tributyrin promotes intestinal development by providing energy for intestinal epithelial cells. Our study extends this conclusion to young pigeons. In the present experiment, dietary tributyrin supplementation significantly increased the jejunal VH and VH/CD of young pigeons, reduced jejunal CD, and simultaneously increased the ileal VH/CD. This indicates that short-chain fatty acids and their derivatives contribute to maintaining intestinal integrity. Dietary supplementation with tributyrin provides a new energy source for intestinal epithelial cells, which to a certain extent promotes the development of small intestinal villi, improves the intestinal morphological structure, and thereby enhances the small intestine’s ability to absorb and utilize nutrients. This finding is closely associated with the significant improvement in the growth performance of young pigeons observed in the preliminary research of this experiment.

### 4.5. The Effect of Tributyrin on the Metabolic Profile of Intestinal Contents in Young Pigeons

#### 4.5.1. The Effect of Tributyrin on Lipid Metabolism in Young Pigeons

Lipids are important cellular components that can provide energy for the body and participate in various life activities [39]. L-carnitine, a key nutrient involved in lipid metabolism [40], can disrupt the gut microbial balance in rats when present in excess: it promotes the proliferation of anaerobic bacteria such as Prevotella and reduces the number of protective bacteria (e.g., the S24_7 group of Bacteroidetes) [41], ultimately leading to liver damage. Additionally, the accumulation of its metabolite trimethylamine N-oxide (TMAO) increases the risk of fatty liver [42]. Myriocin, a thermophilic yeast-inhibiting substance, inhibits serine palmitoyltransferase to block sphingolipid synthesis, thereby reducing the production of neuroceramide in the body and inducing inflammation [43,44]. Phytanic acid, a branched-chain fatty acid, is mainly metabolized through the peroxisomal α-oxidation pathway [45,46]. When related metabolic enzymes are deficient, phytanic acid accumulates in large quantities in the body, causing neurodegenerative diseases [47]; it can also damage vascular smooth muscle cells, and high concentrations of phytanic acid can affect body growth and development [48]. Our results revealed that tributyrin downregulated several harmful lipid metabolites in the intestine of young pigeons.

The results of this study showed that dietary supplementation with 1500 mg/kg tributyrin (TB) significantly reduced the relative contents of L-carnitine, myriocin, and phytanic acid in the small intestine of young pigeons. Specifically, the downregulation of L-carnitine directly reduced the production of pro-inflammatory metabolites such as TMAO, which in turn decreased serum TNF-α, IL-6, and ALT levels, thereby improving immune function and liver health. The coordinated regulation of these key differential metabolites can effectively reduce the accumulation of pro-inflammatory and liver-damaging microbial communities in the intestine and lower the risk of liver function damage, lipid metabolism disorders, and harmful metabolite accumulation, while optimizing the intestinal flora structure, maintaining the body’s microecological balance, alleviating systemic inflammatory responses and oxidative stress, improving immune health status, and collectively protecting the health of young pigeons. These changes comprehensively explain the mechanism by which tributyrin improves lipid metabolism and reduces inflammation in young pigeons.

#### 4.5.2. The Effect of Tributyrin on Amino Acid Metabolism in Young Pigeons

Amino acids are essential raw materials for protein synthesis, providing a material basis for animal growth, metabolism, and life maintenance [49]. They can be converted into carbohydrates, lipids, and various bioactive substances. N-formylkynurenine (NFK), a key intermediate product of tryptophan metabolism, has its metabolic direction regulated by blood hydrolase. When hydrolase is sufficient, NFK is converted into kynurenine (KYN) [50]. KYN participates in immune regulation; when hydrolase is insufficient, N-formylkynurenine-carboxyketene (NFK-CKA) is generated by combining with nucleophilic molecules [51]. Studies have shown that NFK possesses multiple physiological functions, including regulating oxidative balance and delaying tissue aging [52].

7,8-dihydrobiopterin is the natural oxidation product of tetrahydrobiopterin (BH_4_) [53]. It reduces the synthesis efficiency of L-dopa by inhibiting tyrosine hydroxylase activity, thereby affecting the production of key neurotransmitters such as dopamine and interfering with physiological processes including neural regulation [54]. Pyridoxamine, the active form of vitamin B6, is a key cofactor for numerous amino acid metabolic enzymes. It not only indirectly regulates glycolipid metabolism but also exerts tissue-protective effects by inhibiting the formation of advanced glycation end products/advanced lipoxidation end products (AGEs/ALEs) [55]. Studies have demonstrated that pyridoxamine has multiple benefits, including improving metabolic function, exerting anti-inflammatory effects, and protecting liver microcirculation, which can reduce fat accumulation, ameliorate glycolipid disorders, and decrease alanine aminotransferase (ALT) activity and pro-inflammatory factor levels. More importantly, pyridoxamine directly enhances glutathione peroxidase (GSH-Px) activity and total antioxidant capacity (T-AOC) and promotes the synthesis of immunoglobulin G (IgG), establishing a direct mechanistic link between pyridoxamine and the improved immune and antioxidant function observed in young pigeons [56].

N-acetylornithine is a key endogenous intermediate in arginine metabolism [57], and its abnormal increase can reduce the glomerular filtration rate and increase the risk of chronic kidney disease [58]. Indoleacetic acid (IAA), an important metabolite of tryptophan, can act as a potent agonist of the aromatic hydrocarbon receptor (AhR) [59]. It exerts neuroprotective and anti-inflammatory effects through the AhR/RAGE/NF-κB signaling pathway, inhibiting microglial cell activation and pro-inflammatory factor release, thereby reducing tissue damage [60]. Trp-Thr-His, as functional amino acids, play a key role in animal metabolism [61]; they can regulate the body’s immune response and maintain the antioxidant capacity of the intestinal barrier [62].

Studies have shown that under Salmonella infection conditions, Trp-Thr-His can significantly improve the physiological state of growing pigs [63] and reduce bacterial adhesion by promoting mucin synthesis [64]. The indole derivatives produced by Trp-Thr-His metabolism can inhibit pathogen colonization [65] and regulate the immune response in growing pig models to alleviate oxidative stress. This synergistic effect can lower rectal temperature, improve fecal scores, and optimize amino acid metabolic balance in pigs [66].

Our metabolomic results revealed that tributyrin enhanced beneficial amino acid and vitamin metabolism. In this experiment, the relative contents of N-methylcysteine, pyridoxamine, tryptophan, threonine, histidine, and indoleacetic acid in the small intestine of young pigeons in the B group (tributyrin treatment group) were significantly increased. This may be related to the role of tributyrin (TB) in maintaining the body’s metabolic homeostasis and inhibiting inflammatory responses, which is consistent with the significant improvement in the immune performance of young pigeons observed in this study. These metabolic changes provide a mechanistic explanation for the enhanced immunity and antioxidant capacity observed in the present study.

#### 4.5.3. The Effects of Tributyrin on Other Metabolites of Young Pigeons

Porphyrin can induce the expression of heme oxygenase-1 (HO-1) and promote heme degradation, thereby exerting specific physiological functions [67]. However, as the dose of this substance increases, it consumes the content of cytochrome P450 enzymes in the liver, thereby impairing the body’s metabolic detoxification capacity and increasing the risk of toxic damage [68,69]. (7E, 13Z, 18R, 20Z)-felixinin exerts antioxidant activity by protecting DNA from oxidative damage and possesses both anti-cancer and cancer-preventive potential [70]. Allyl methyl sulfoxide (AMSO_2_), as an active metabolite of garlic, exerts a protective effect on animal kidneys by inhibiting the generation of reactive oxygen species (ROS) [71], blocking the mitogen-activated protein kinase/nuclear factor-κB (MAPK/NF-κB) signaling pathway, and regulating apoptosis-related proteins [72].

Collectively, these changes in metabolites form a coordinated regulatory network that supports intestinal and liver health. The results of this experiment showed that the relative contents of (7E, 13Z, 18R, 20Z)-filicinin and allyl methyl sulfoxide in the small intestine of young pigeons in group B (tributyrin treatment group) were significantly increased. This may be associated with the role of tributyrin (TB) in enhancing the body’s antioxidant capacity, reducing oxidative stress damage and improving immune function and overall health status. Meanwhile, TB significantly reduced the relative content of protoporphyrin IX in the small intestine of young pigeons, which helps alleviate liver metabolic dysfunction, reduce liver toxicity, and lower the risk of related metabolic disorders.

## 5. Conclusions

Based on the present results, dietary supplementation with 1500 mg/kg tributyrin effectively improved lipid metabolism, immune function, antioxidant capacity, intestinal morphology, and intestinal metabolic homeostasis in young pigeons.

Specifically, tributyrin significantly decreased serum triglyceride content and alanine aminotransferase activity, while increasing high-density lipoprotein concentration, suggesting improved lipid metabolism and liver health. Meanwhile, serum immunoglobulin G content was significantly increased, and pro-inflammatory cytokines including TNF-α and IL-6 were decreased, indicating enhanced humoral immunity and suppressed excessive inflammatory response. In addition, serum total antioxidant capacity and glutathione peroxidase activity were highly significantly improved, revealing strengthened antioxidant defense ability.

Furthermore, tributyrin optimized jejunal and ileal morphology by increasing villus height and villus height/crypt depth ratio, thereby improving intestinal barrier integrity and nutrient absorption. Intestinal metabolomic analysis confirmed that tributyrin regulated key metabolic pathways including 2-oxocarboxylic acid metabolism, tryptophan metabolism, and vitamin B6 metabolism, which were closely associated with anti-inflammatory, antioxidant, and nutrient utilization processes.

In summary, 1500 mg/kg tributyrin exerts comprehensive beneficial effects on young pigeons by regulating lipid metabolism, enhancing immune and antioxidant function, improving intestinal structure, and modulating intestinal metabolic network. These results support tributyrin as a safe and effective green feed additive for improving health status of young meat pigeons and provide a scientific basis for its application in antibiotic-free pigeon breeding.

## Figures and Tables

**Table 1:** Composition and nutritional levels of the basal diet (air-dry basis) %.

Items	Contents
Raw grain	
Corn	40.00
Sorghum	5.00
Pea	10.00
Wheat	5.00
Pellets	
Corn	19.80
Soybean meal	12.60
Wheat bran	0.80
Limestone powder	0.80
Sodium bicarbonate	0.40
Salt	0.40
Yeast powder	2.00
Calcium hydrogen phosphate	0.60
Methionine	0.16
Lysine	0.24
Soybean oil	0.40
Premix ^①^	1.80
Total	100.00
Nutritional levels ^②^	
Metabolizable energy (MJ/kg)	12.00
Crude protein	16.00
Lysine	1.00
Methionine	0.40
Methionine + cystine	0.80
Tryptophan	0.20
Threonine	0.60
Calcium	1.30
Available phosphorus	0.35
Salt	0.40

Note ^①^: Each kilogram of premix provides the following nutrients for the diet: vitamin A 27,000 IU, vitamin D_3_ 90,000 IU, vitamin E 850 mg, vitamin K_3_ 92 mg, vitamin B_2_ 160 mg, vitamin B_6_ 115 mg, niacinamide 1050 mg, D-calcium pantothenate 520 mg, D-biotin 2 mg, folic acid 20 mg, Cu 340 mg, Fe 1700 mg, Mn 1700 mg, Zn 1400 mg; ^②^: all nutritional levels are calculated values.

**Table 2:** Gradient elution program of chromatography.

Time Min	Mobile Phase A/%	Mobile Phase B/%	Flow Rate (mL/min)
0	98	2	0.20
1.5	98	2	0.20
3	15	85	0.20
10	0	100	0.20
10.1	98	2	0.20
11	98	2	0.20
12	98	2	0.20

**Table 3:** Effects of tributyrin on serum biochemical indicators of young pigeons.

Item	CK Group	B Group	*p*-Value
TP (g/L)	29.12 ± 2.21	29.45 ± 1.66	0.774
ALB (g/L)	10.68 ± 1.33	10.63 ± 1.06	0.944
TC (mmol/L)	6.90 ± 0.54	7.15 ± 0.66	0.488
TG (mmol/L)	0.81 ± 0.06 ^a^	0.66 ± 0.10	0.011
HDL (mmol/L)	3.13 ± 0.38	3.76 ± 0.39 ^a^	0.018
LDL (mmol/L)	0.85 ± 0.22	0.65 ± 0.13	0.086
ALT (U/L)	16.98 ± 2.81 ^A^	11.03 ± 1.43	0.001
AST (U/L)	82.00 ± 13.90	75.85 ± 18.54	0.530

Note: ^a^, significant difference (*p* < 0.05); ^A^, highly significant difference (*p* < 0.01).

**Table 4:** Effects of tributyrin on serum immune parameters of young pigeons.

Item	CK Group	B Group	*p*-Value
IgG (ug/mL)	14.07 ± 2.45	19.00 ± 1.81 ^A^	0.003
IgM (ug/mL)	2.12 ± 0.49	2.41 ± 0.69	0.427
IgA (pg/mL)	62.46 ± 8.98	67.76 ± 10.51	0.368
IL-1 (pg/mL)	17.08 ± 3.93	17.07 ± 4.92	0.998
TNF-α (pg/mL)	4.27 ± 0.62 ^a^	3.53 ± 0.41	0.036
IL-1β (pg/mL)	8.46 ± 2.07	7.90 ± 1.72	0.622
IL-6 (pg/mL)	12.25 ± 1.11 ^a^	9.67 ± 1.69	0.011

Note: ^a^, significant difference (*p* < 0.05); ^A^, highly significant difference (*p* < 0.01).

**Table 5:** Effects of tributyrin on serum antioxidant indicators of young pigeons.

Item	CK Group	B Group	*p*-Value
T-AOC (U/mL)	3.26 ± 1.38	6.13 ± 1.88 ^A^	0.004
GSH-Px (U/mL)	172.98 ± 19.88	211.17 ± 17.91 ^A^	0.001
MDA (nmol/mL)	5.01 ± 1.94	4.37 ± 0.75	0.399
T-SOD (U/mL)	87.84 ± 5.12	88.99 ± 4.60	0.643

Note: ^A^, highly significant difference (*p* < 0.01).

**Table 6:** Effects of tributyrin on intestinal morphology of young pigeons.

Item		CK Group	B Group	*p*-Value
Duodenum	VH (μm)	941.18 ± 63.48	1006.27 ± 71.33	0.075
	CD (μm)	185.01 ± 28.63	194.19 ± 20.35	0.473
	VH/CD	5.17 ± 0.69	5.24 ± 0.73	0.849
Jejunum	VH (μm)	539.63 ± 40.8	589.27 ± 42.79 ^a^	0.032
	CD (μm)	129.54 ± 13.18 ^A^	103.08 ± 8.37	<0.001
	VH/CD	4.19 ± 0.41	5.74 ± 0.56 ^A^	<0.001
Ileum	VH (μm)	295.20 ± 29.66	319.96 ± 19.84	0.073
	CD (μm)	93.76 ± 18.96 ^a^	73.95 ± 11.29	0.027
	VH/CD	3.26 ± 0.73	4.40 ± 0.62 ^A^	0.005

Note: ^a^, significant difference (*p* < 0.05); ^A^, highly significant difference (*p* < 0.01).

**Table 7:** List of differential metabolites in small intestinal contents of young pigeons (positive ion mode).

Differential Metabolites	RT (min)	m (z)	VIP	FC	*p*-Value	Changetrend
Thiobenzamide S	5.85	170.03	2.59	3.44	<0.001	Upregulated
Protoporphyrin IX	9.67	563.27	2.55	0.11	<0.001	Downregulated
(7E,13Z,18R,20Z)-felixinin	8.30	411.26	2.06	4.86	0.003	Upregulated
Allyl methyl sulfone	0.93	121.03	1.83	1.59	0.012	Upregulated
L-carnitine	1.44	162.11	1.93	0.72	0.014	Downregulated
N’-formylkynurenine	4.97	237.09	1.82	4.39	0.017	Upregulated
Myriocin	6.69	384.27	1.84	0.38	0.017	Downregulated
7,8-dihydrobiopterin	2.27	240.11	1.77	0.45	0.022	Downregulated
Trp-Thr-His	8.29	443.20	1.59	1.88	0.032	Upregulated
Pyridoxamine	0.62	169.10	1.50	1.49	0.045	Upregulated

**Table 8:** List of differential metabolites in small intestinal contents of young pigeons (negative ion mode).

Differential Metabolites	RT (min)	m (z)	VIP	FC	*p*-Value	Changetrend
N-acetylornithine	2.00	173.09	2.05	0.37	0.007	Downregulated
Indoleacetic acid	4.97	174.06	1.96	3.56	0.010	Upregulated
Phytanic acid	11.54	311.30	1.70	0.34	0.026	Downregulated

## Data Availability

The raw datasets generated and analyzed during the current study are not publicly available due to institutional internal data management requirements. The supporting data that confirm the findings of this work are available from the corresponding author upon reasonable request.

## References

[1] Wang Y.R., Ma J., Yang C.S., Wang L.Y. (2026). Situation, problems, challenges and countermeasures of meat pigeon industry at home and abroad. Chin. J. Anim. Sci..

[2] Xu S.J., Zhao K.L., Li J., Chen Y.F., Zhang B., Fu Y.Q., Chen H. (2026). Research progress on main nutritional requirements of breeding pigeons at different physiological stages. Feed Ind..

[3] Li H., Zhang J., Li H., Li X., Zhang P., Guo X., Lin J., Liao K., Ke L. (2025). Effect of *Portulaca oleracea* Addition in Health Care Sand on Apparent Nutrient Digestibility, Serum Parameters, and Excreta Microbiota Metabolism in Tumbler Pigeons. Animals.

[4] Yan Y., Yang Z., Yang H. (2024). Function of tributyrin and its application research progress in livestock and poultry production. Feed Ind..

[5] Liang Y., Fan Y., Zhou X., Wan Y., Yu W., You P., Yu X., Shi X., Deng K., Wang F. (2025). Effects of dietary tributyrin supplementation on growth performance, slaughter performance, meat quality and serum indices of Hu sheep. J. Nanjing Agric. Univ..

[6] Lei X., Shi L., Shao M., Peng Y., Shen Z., Zhao K. (2024). Effects of *Nauclea officinalisand* tributyrin on growth performance, immune function and intestinal flora of Wenchang chickens. China Feed.

[7] Peng L., Sun J., Shi Y., Zhu G., Li W., Yu D. (2014). Effects of tributyrin on growth performance, nutrient apparent digestibility, slaughter performance, intestinal morphology and microbial flora of broilers. Chin. J. Anim. Nutr..

[8] Chen G., Zhuo R., Ding H., Yang K., Xue J., Zhang S., Chen L., Yin Y., Fang R. (2022). Effects of dietary tributyrin and phytosterol ester supplementation on growth performance, intestinal morphology, microbiota and metabolites in weaned piglets. J. Appl. Microbiol..

[9] Zhou G., Tao Y., Yin J., Ni L., Zhao X., Wang B. (2023). Effects of *Tenebrio molitormeal* on growth performance, serum biochemical indices and hormone levels of finishing pigs. Chin. J. Anim. Nutr..

[10] Liu L., Ma W., Li L., Yuan C., Shi Z., Liu Y., Qin R., Wang W. (2025). Effects of fermented Chinese herbal medicine on growth performance, serum biochemistry and growth hormone of lambs. Xinjiang Agric. Sci..

[11] Liu J., Guo R., Zhang J., Zhang L., Yang W., Xin G. (2024). Effects of energy and protein levels on growth performance, slaughter performance, blood biochemical indices and metabolomics of Jingyuan chickens. Southwest China J. Agric. Sci..

[12] Xu Z. (2022). Effects of Dietary Energy and Protein on Production Performance, Intestinal Microbiota and Lipid Metabolism Gene Expression of Jingdian Beijing Ducks. Master’s Thesis.

[13] Zhou X., Xu J., Zeng Y., Zhu H., Wu S., Shao C., Cheng G., Shu Y. (2025). Effects of fermented Yupingfeng powder on laying performance, egg quality, serum biochemical and antioxidant indices of Roman laying hens. Feed Res..

[14] Wang X. (2022). Effects of Combined Protease on Feeding Preference, Blood Biochemical Indices and Intestinal Morphology of Broilers. Master’s Thesis.

[15] Jin H., Zhao D., Du X., Shan Y., Liu F. (2022). Effects of tributyrin and oxymatrine on growth performance, serum biochemical indices and intestinal health of broilers. Anim. Husb. Vet. Med..

[16] Lu W., Yang H., Chen Z., Cao Y., Wang M., Yin S., Wang Y. (2024). Effects of dietary tributyrin supplementation on growth performance, nutrient digestibility and serum biochemical indices of broilers. China Feed..

[17] Yang L. (2012). Effects of tributyrin on growth performance, intestinal structure and blood biochemical indices of weaned piglets. Feed Ind..

[18] Feng Y., Zhao J., Bian B., Wei Y., Li J., Guo X., Li Y. (2025). Polygonum hydropiper improves fermentation quality of wet-stored corn and enhances resistance of chicks to Salmonella infection. Chin. J. Anim. Sci..

[19] Yang C., Wang S., Li Q., Zhang R., Xu Y., Feng J. (2024). Effects of probiotic *Lactiplantibacillus plantarum* HJLP-1 on growth performance, selected antioxidant capacity, immune function indices in the serum, and cecal microbiota in broiler chicken. Animals.

[20] Xu J., Wu Z., Peng S., Yu Y., Lin F., Ma F., Chen R., Wang X., Li Z. (2025). Effects of a novel Chinese herbal compound preparation on growth performance, serum immune indices and intestinal flora of broilers. Anim. Husb. Vet. Med..

[21] Feng Z., Li L., Liu M., Zhang X., Li X., Lu Y., Chen Y. (2023). Effects of sea buckthorn pomace extract on growth performance, antioxidant capacity and immune function of yellow-feathered broilers under oxidative stress. Chin. J. Anim. Nutr..

[22] Xie Z., Wang J. (2025). Effects of apigenin on production performance, egg quality and serum immune indices of laying hens in late laying period. China Feed.

[23] Zhang G. (2024). Effects of Macleaya Cordata extract on Production Performance, Immune Function and Intestinal Microbiota of Broilers and Laying Hens. Ph.D. Thesis.

[24] Liu X. (2020). Effects of coated sodium butyrate on growth performance, immune function and intestinal morphology of broilers. Feed Res..

[25] Miao R., Wu K., Sun Y., Li W., Wang Y., Qu Y. (2024). Effects of plant essential oil complex tributyrin on growth performance, blood indices and intestinal morphology of mice challenged with *Escherichia coli*. Chin. J. Anim. Nutr..

[26] Tong N., Chen Y., Liu H., Wang X., Qi X., Bai M., Liu Z., Peng L. (2025). Evaluation of medicinal quality of tree peony byproducts and the effects of dietary byproducts supplementation on production performance, egg quality, serum antioxidant levels, and gut microbiota in late-phase laying hens. Poult. Sci..

[27] Surai P.F., Kochish I.I., Fisinin V.I., Kidd M.T. (2019). Antioxidant defence systems and oxidative stress in poultry biology: An update. Antioxidants.

[28] Chen W., Shi P., Li Y., Hou Z., Li H. (2024). Research progress of superoxide dismutase in animal production. Feed Res..

[29] Wu D. (2024). Construction of Catalase Nanocapsules and Their Application in the Treatment of Acute Liver Injury. Ph.D. Thesis.

[30] Lü Y. (2019). Effects of Malondialdehyde Oxidative Stress on Emulsification and Gel Properties of Pork Myofibrillar Protein. Master’s Thesis.

[31] Wang J., Zhang H., Bai S., Zeng Q., Su Z., Zhuo Y., Mao X., Yin H., Feng B., Liu J. (2021). Dietary tributyrin improves reproductive performance, antioxidant capacity, and ovary function of broiler breeders. Poult. Sci..

[32] Wang C., Cao S., Zhang Q., Shen Z., Feng J., Hong Q., Lu J., Xie F., Peng Y., Hu C. (2019). Dietary tributyrin attenuates intestinal inflammation, enhances mitochondrial function and induces mitophagy in piglets challenged with diquat. J. Agric. Food Chem..

[33] Xu J., Zhang Z., Wang D., Chen Y., Liu X., Zhang H., Huang C., Liu M., Zhang B., Hou J. (2021). Effects of garlic essential oil in drinking water on growth performance, intestinal morphology and cecal microbiota of laying hens. Chin. J. Anim. Nutr..

[34] Li W., Wei K., Zhang S., Chen Y. (2021). Effects of walnut green husk and its extracts on intestinal morphology, mucosal antioxidant properties and microbial diversity of yellow-feathered broilers. Chin. Anim. Husb. Vet. Med..

[35] Liang Y., Xu X., Tang J., Xu B., Zhang L., Huang W., Du Z., Li Y. (2025). Effects of Lactobacillus supplementation in drinking water on production performance, intestinal morphology, cecal microbiota structure and serum antioxidant indices of broiler breeders. Chin. J. Anim. Sci..

[36] Ismael E., Kamel S., Elleithy E.M.M., Bekeer M.R., Fahmy K.N.E. (2025). Comparative effects of dietary sodium butyrate and tributyrin on broiler chickens’ performance, gene expression, intestinal histomorphometry, blood indices, and litter. Sci. Rep..

[37] Shang Y. (2024). Application of Tributyrin and Glycerol Monolaurate Mixture in Broilers. Master’s Thesis.

[38] Cui L., Yuan Y., Quan Z., Yan J. (2009). Effects of different levels of sodium butyrate on intestinal morphology of broilers. Feed Ind..

[39] He T., Liu C., Li W., Tian Y., Wang W., Yuan F., Chen S., Zhong K., Huang J. (2020). Meta-analysis bridging network pharmacology for clinical efficacy evaluation of Huanglian Ejiao Decoction in treating type 2 diabetes and preliminary exploration of its potential mechanism. Chin. Tradit. Herb. Drugs.

[40] Wu Q., Zhang X.N., Zhao Y., Yang X.B. (2020). High l-Carnitine Ingestion Impairs Liver Function by Disordering Gut Bacteria Composition in Mice. J. Agric. Food Chem..

[41] Tang W.J., Yao X.R., Xia F., Yang M.T., Chen Z.J., Zhou B.J., Liu Q. (2018). Modulation of the Gut Microbiota in Rats by Hugan Qingzhi Tablets During the Treatment of High-Fat-Diet-Induced Nonalcoholic Fatty Liver Disease. Oxidative Med. Cell. Longev..

[42] Zhu Y.J., Jameson E., Crosatti M., Schäfer H., Rajakumar K., Bugg T.D.H., Chen Y. (2014). Carnitine metabolism to trimethylamine by an unusual Rieske-type oxygenase from human microbiota. Proc. Natl. Acad. Sci. USA.

[43] Edukulla R., Rehn K.L., Liu B., McAlees J.W., Hershey G.K., Wang Y.H., Lewkowich L., Lindsley A.W. (2016). Intratracheal myriocin enhances allergen-induced Th2 inflammation and airway hyper-responsiveness. Immun. Inflamm. Dis..

[44] Wadsworth J.M., Clarke D.J., McMahon S.A., Lowther J.P., Beattie A.E., Langridge-Smith P.R.R., Broughton H.B., Dunn T.M., Naismith J.H., Campopiano D.J. (2013). The chemical basis of serine palmitoyltransferase inhibition by myriocin. J. Am. Chem. Soc..

[45] Wanders J.R., Komen J., Ferdinandusse S. (2011). Phytanic acid metabolism in health and disease. Biochim. Biophys. Acta Mol. Cell Biol. Lipids.

[46] Neha, Chaudhary S., Tiwari P., Parvez S. (2024). Amelioration of Phytanic Acid-Induced Neurotoxicity by Nutraceuticals: Mechanistic Insights. Mol. Neurobiol..

[47] Busanello N.E.B., Amaral A.U., Tonin A.M., Zanatta A., Viegas C.M., Vargas C.R., Wajner M. (2013). Disruption of mitochondrial homeostasis by phytanic acid in cerebellum of young rats. Cerebellum.

[48] Leipnitz G., Amaral U.A., Zanatta Â., Seminotti B., Fernandes C.G., Knebel L.S., Vargas C.R., Wajner M. (2010). Neurochemical evidence that phytanic acid induces oxidative damage and reduces the antioxidant defenses in cerebellum and cerebral cortex of rats. Life Sci..

[49] Xiao Y.P., Wu T.X., Hong Q.H., Sun J.M., Chen A.G., Yang C.M., Li X.Y. (2012). Response to weaning and dietary L-glutamine supplementation: Metabolomic analysis in piglets by gas chromatography/mass spectrometry. J. Zhejiang Univ. Sci. B.

[50] Purwani N.N., Rozeboom J.H., Willers P.V., Wijma H.J., Fraaije M.W. (2024). Discovery of a new class of bacterial heme-containing C=C cleaving oxygenases. New Biotechnol..

[51] Li B., Kim J.Y., Martis E.M., Donaldson P.J., Lim J.C. (2020). Characterisation of Glutathione Export from Human Donor Lenses. Transl. Vis. Sci. Technol..

[52] Wang Y., Leung E., Tomek P. (2025). N-formylkynurenine but not kynurenine enters a nucleophile-scavenging branch of the immune-regulatory kynurenine pathway. Bioorganic Chem..

[53] Xu Y., Li Y., Li L., Zhang L., Ding Z., Shi G. (2021). Reductase-catalyzed tetrahydrobiopterin regeneration alleviates the anti-competitive inhibition of tyrosine hydroxylation by 7,8-dihydrobiopterin. Catal. Sci. Technol..

[54] Dunkley P.R., Dickson P.W. (2019). Tyrosine hydroxylase phosphorylation In Vivo. J. Neurochem..

[55] Wilson P.M., Plecko B., Mills B.P., Clayton T.P. (2019). Disorders affecting vitamin B_6_ metabolism. J. Inherit. Metab. Dis..

[56] Silvares R.R., Araujo D.P.B., Pereira E.N.G.D.S., Rodrigues K.L., Barbosa J.M.C., Silva J.F.D., Silva V.V.D., Aarenburg M.V.D., Scheijen J., Wouters K. (2026). Pyridoxamine reduces inflammatory and microcirculatory abnormalities in metabolic dysfunction-associated steatohepatitis and modulates key factors in the hepatic AGE/ALE signaling pathway. Front. Physiol..

[57] Luo S.Y., Surapaneni A., Zheng Z., Rhee E.P., Coresh J., Hung A.M., Nadkarni G.N., Yu B., Boerwinkle E., Tin A. (2020). NAT8 Variants, N-Acetylated Amino Acids, and Progression of CKD. Clin. J. Am. Soc. Nephrol..

[58] Liu Y.Q., Ling L.L., Shen Y., Bi X. (2024). Metabolome-wide Mendelian randomization reveals causal effects of betaine and N-acetylornithine on impairment of renal function. Front. Nutr..

[59] Wang N., Sun C.Y., Yang Y.J., Zhang D.D., Huang L.L., Xu C.R., Wang M., Xu M., Yan T., Wu Y. (2025). Gut microbiota-derived indoleacetic acid attenuates neuroinflammation and neurodegeneration in glaucoma through ahr/rage pathway. J. Neuroinflamm..

[60] Tintelnot J., Xu Y., Lesker R.T., Schönlein M., Konczalla L., Giannou A.D., Pelczar P., Kylies D., Puelles V.G., Bielecka A.A. (2025). Microbiota-derived 3-IAA influences chemotherapy efficacy in pancreatic cancer. Nature.

[61] Yu Z.M., Hoffmann A., Irion L.A., Ram M., Drozak J., Rentmeister A., Mecinović J. (2026). Histidine methylation via an enzymatic cascade with in situ generation of nucleoside-modified AdoMet analogues. Chem. Commun..

[62] Chalvon-Demersay T., Luise D., Floc’h N.L., Tesseraud S., Lambert W., Bosi P., Trevisi P., Beaumont M., Corrent E. (2021). Functional Amino Acids in Pigs and Chickens: Implication for Gut Health. Front. Vet. Sci..

[63] Valini C.D.A.G., Arnaut R.P., França I., Ortiz M.T., Oliveira M.J.K., Melo A.D.B., Marçal D.A., Campos P.H.R.F., Htoo J.K., Brand H.G. (2023). Increased dietary Trp, Thr, and Met supplementation improves growth performance and protein deposition of salmonella-challenged growing pigs under poor housing conditions. J. Anim. Sci..

[64] Campos H.R.P., Merlot E., Damon M., Noblet J., Floc’h N.L. (2014). High ambient temperature alleviates the inflammatory response and growth depression in pigs challenged with *Escherichia coli* lipopolysaccharide. Vet. J..

[65] McGilvray W.D., Wooten H., Rakhshandeh A.R., Petry A., Rakhshandeh A. (2019). Immune system stimulation increases dietary threonine requirements for protein deposition in growing pigs. J. Anim. Sci..

[66] Rodrigues A.L., Wellington O.M., González-Vega C.J., Htoo J.K., Kessel A.G.V., Columbus D.A. (2021). Functional amino acid supplementation, regardless of dietary protein content, improves growth performance and immune status of weaned pigs challenged with Salmonella Typhimurium. J. Anim. Sci..

[67] Bednarz A., Kożuch P., Kowalski K., Skulimowska I., Kachamakova-Trojanowska N., Filipek-Gorzała J., Kwiecińska P., García-García R., Gawlińska K., Mależyna K. (2025). Cobalt protoporphyrin IX induces transient, dose- and time-dependent granulocyte mobilization with mild metabolic effects in mice. Pharmacol. Rep..

[68] Muhoberac B.B., Hanew T., Halter S., Schenker S. (1989). A model of cytochrome P-450-centered hepatic dysfunction in drug metabolism induced by cobalt-protoporphyrin administration. Biochem. Pharmacol..

[69] Deodhar M., Al Rihani S.B., Arwood M.J., Darakjian L., Dow P., Turgeon J., Michaud V. (2020). Mechanisms of CYP450 Inhibition: Understanding Drug-Drug Interactions due to Mechanism-Based Inhibition in Clinical Practice. Pharmaceutics.

[70] Jiang H.Y., Ahn Y.E., Ryu H.S., Kim D.K., Park J.S., Kang S.W., You S., Lee B.J., Jung J.H. (2005). Antioxidant Activity of (8E,13Z,20Z)-Strobilinin/(7E,13Z,20Z)-Felixinin from a Marine Sponge *Psammocinia* sp.. Nat. Prod. Sci..

[71] Meng X.M., Ren G.L., Gao L., Yang Q., Li H.D., Wu W.F., Huang C., Zhang L., Lv X.W. (2018). NADPH oxidase 4 promotes cisplatin-induced acute kidney injury via ROS-mediated programmed cell death and inflammation. Lab. Investig..

[72] Yu H., Lin L.B., Zhang Z.Q., Zhang H.Y., Hu H.B. (2020). Targeting NF-κB pathway for the therapy of diseases: Mechanism and clinical study. Signal Transduct. Target. Ther..

